# Edge Effects on Carbon, Nitrogen, Phosphorus, and Non-Structural Carbohydrates in Organs of *Salsola collina*

**DOI:** 10.3390/plants15192979

**Published:** 2026-09-29

**Authors:** Yingjie Gao, Yonggang Li, Dongxiu Duan, Xiaoyu Tang, Xiuwen Shen, Mengnan Yi, Bingqian Su, Zhao Fang, Wenlong Xu, Wenwen Huang, Hao Yu

**Affiliations:** School of Plant Protection and Environment, Henan Institute of Science and Technology, Xinxiang 453003, China

**Keywords:** *Salsola collina*, patch edge effect, ecological stoichiometry, non-structural carbohydrates, organ-specific responses

## Abstract

Plant patches are a common feature of dryland ecosystems, but how within-patch spatial position is associated with plant functional traits–and whether these patterns differ among organs—remains poorly understood. We sampled twigs, leaves, and flowers of the C_4_ desert plant *Salsola collina* from the centers and edges of natural patches in arid Xinjiang, China, using five plots with three patches per plot. For each organ, we measured the concentrations of carbon (C), nitrogen (N), phosphorus (P), sucrose (SUC), fructose (FRU), total soluble sugars (SS), and starch (ST), and calculated non-structural carbohydrate (NSC) concentrations as the sum of SS and ST. Treating the plot as the experimental unit and accounting for paired center–edge observations within plots, we evaluated the effects of patch position, organ type, and their interaction using two-way ANOVA and distance-based multivariate analyses. Organ type accounted for the largest proportion of multivariate trait variation (*R*^2^ = 0.90), whereas patch position and the Patch × Organ interaction each explained approximately 4% (all *p* < 0.001). Significant Patch × Organ interactions were detected for 14 of 17 traits, indicating that center–edge differences varied among organs for most traits. N concentrations were higher at patch edges in all three organs, whereas P concentrations were higher in twigs and flowers only; C showed no significant center–edge differences. NSC concentrations were higher at patch edges in all three organs, while SS was higher in edge twigs and leaves and ST was higher in edge flowers. These patterns were accompanied by contrasting organ-specific differences in carbohydrate composition and elemental stoichiometry. Because environmental variables were not measured concurrently, the observed patterns should be interpreted as spatial associations rather than demonstrated environmental mechanisms. These findings indicate that organ identity and within-patch position are jointly associated with variation in the carbon-, nutrient-, and carbohydrate-related traits of *S. collina*, highlighting the importance of considering organ-specific variation when evaluating fine-scale spatial heterogeneity in dryland plants.

## 1. Introduction

In arid regions, water and nutrients are often limited and spatially heterogeneous, and vegetation commonly occurs as discrete patches surrounded by relatively bare soil [1]. Vegetation patches can modify local resource distributions by intercepting water, nutrients, and sediments, thereby generating fine-scale spatial heterogeneity within and around patches [2,3]. Differences between patch centers and edges have been reported for vegetation and soil properties [4]. In the present study, the term patch edge effect refers specifically to differences in plant traits associated with plant position at the patch center versus the patch edge, rather than to a demonstrated environmental mechanism. *Salsola collina* Pall. is a C_4_ desert species commonly distributed in arid and semi-arid regions of northwestern China [5]. Previous studies on this species have mainly focused on its responses to abiotic stress, including leaf anatomical and physiological characteristics, transcriptional responses [5,6], and seed germination ecology [7,8]. However, whether plant functional traits differ consistently between individuals growing at different positions within naturally occurring *S. collina* patches remains poorly understood.

Vegetation patches may exhibit substantial internal environmental heterogeneity. Patch centers and edges can potentially differ in resource availability, light conditions, microclimate, neighboring vegetation, and plant–soil interactions [2,3,4]. Such fine-scale variation may be associated with differences in plant tissue traits even within the same species. However, the direction and magnitude of center–edge differences are likely to vary among ecosystems, species, and plant organs. Importantly, these environmental factors were not directly measured in the present study and therefore represent possible ecological contexts rather than demonstrated drivers of plant trait variation. A key unresolved question is whether nutrient and carbohydrate traits of *S. collina* differ between patch centers and edges and whether the magnitude or direction of these differences varies among twigs, leaves, and flowers.

Plant ecological stoichiometry, commonly characterized by tissue carbon (C), nitrogen (N), and phosphorus (P) concentrations and their ratios, provides information on tissue nutrient status and elemental composition. Previous studies have demonstrated substantial organ-specific variation in plant N and P concentrations and stoichiometric relationships [9,10]. In arid regions of northwestern China, reproductive and vegetative organs may differ markedly in N and P concentrations [11], while nutrient stoichiometry can also vary among leaves, stems, and roots within related plant taxa [12]. Differences in tissue composition are associated with the contrasting structural, photosynthetic, and reproductive functions of plant organs [13]. At the same time, fine-scale spatial heterogeneity within vegetation patches may be associated with changes in tissue nutrient concentrations, although such patterns may differ among organs. Therefore, examining C, N, and P concentrations and their stoichiometric ratios across twigs, leaves, and flowers can help determine whether organ-specific nutrient composition is associated with plant position within *S. collina* patches.

Non-structural carbohydrates (NSC), mainly soluble sugars and starch, constitute an important pool linking recently assimilated carbon with short-term use and storage [14]. NSC concentrations and their components vary among plant organs [15]. Experimental studies have also shown that NSC-related traits can vary under drought and changes in water and nutrient conditions [16,17,18,19]. These studies provide physiological context, but responses to experimentally imposed drought should not be assumed to represent conditions at natural patch edges. In naturally heterogeneous vegetation patches, differences in local environmental conditions may instead be associated with variation in the concentrations and relative composition of NSC components. In addition to total soluble sugars and starch, individual soluble sugars such as sucrose and fructose can provide complementary information on carbohydrate composition and may show different patterns among organs or spatial positions. Integrating C–N–P stoichiometric traits with NSC and its major components therefore provides a framework for describing how nutrient and carbohydrate traits vary among organs and between patch centers and edges.

Despite increasing knowledge of organ-specific stoichiometry and NSC variation, little is known about whether these two groups of traits show coordinated center–edge differences among different organs within natural desert plant patches. In this study, we compared C, N, and P concentrations and their stoichiometric ratios, together with total soluble sugars, starch, NSC, sucrose, fructose, and related carbohydrate ratios, in twigs, leaves, and flowers of *S. collina* collected from patch centers and edges in arid Xinjiang, China. We addressed three questions: (1) How do ecological stoichiometric and NSC traits differ among twigs, leaves, and flowers? (2) Are these nutrient and carbohydrate traits associated with position within the patch, i.e., patch center versus edge? and (3) Does the direction or magnitude of center–edge differences vary among organs? By addressing these questions, this study characterizes organ-specific trait patterns associated with within-patch spatial position and provides a basis for further investigating the environmental processes underlying fine-scale trait variation in dryland plants. The present study was designed specifically to examine spatial variation within established *S. collina* patches; comparisons with plants occurring outside defined patches were beyond the scope of the original sampling design.

## 2. Results

### 2.1. Effects of Organ Type and Patch Position on Ecological Stoichiometry and Non-Structural Carbohydrate Traits of S. collina

**Figure 1:**
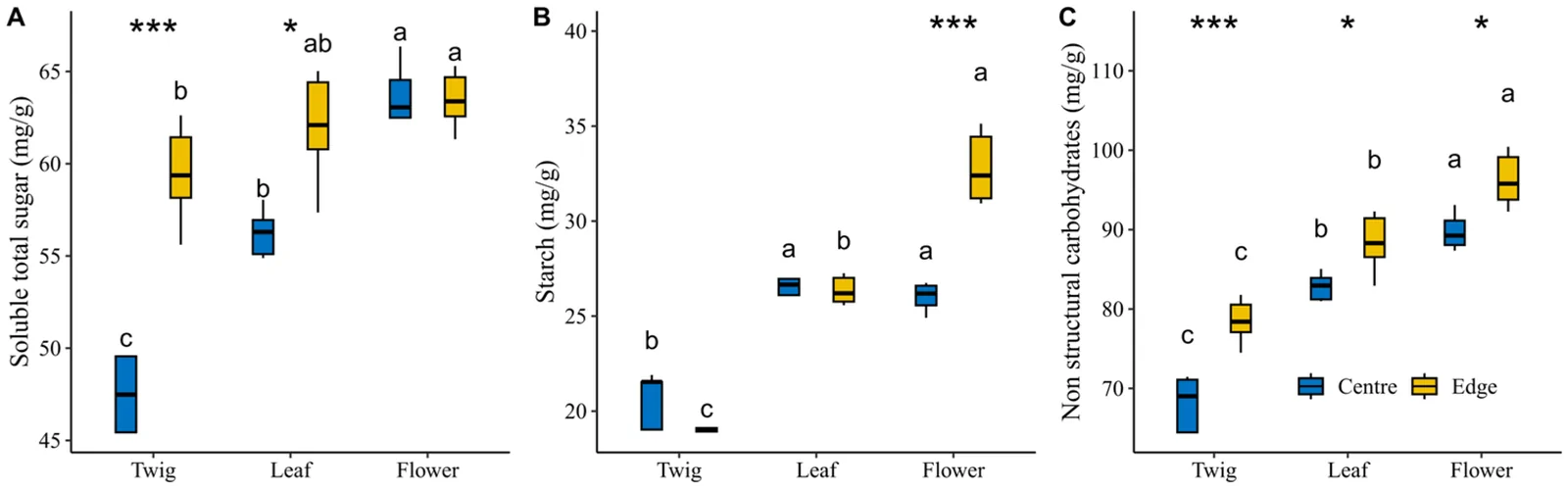
Total soluble sugar, starch, and non-structural carbohydrate concentrations in twigs, leaves, and flowers of *Salsola collina* at patch centers and edges: (**A**) total soluble sugar (SS), (**B**) starch (ST), and (**C**) non-structural carbohydrates (NSC). Note: Data are based on five plot-level replicates (*n* = 5). Lowercase letters compare organs within the same patch position. Groups sharing at least one letter (e.g., a and ab) do not differ significantly, whereas groups with no letters in common differ significantly (*p* < 0.05). Asterisks compare patch centers and edges within the same organ: one and three asterisks indicate * *p* < 0.05 and *** *p* < 0.001, respectively. The absence of an asterisk indicates no significant center–edge difference.

**Figure 2:**
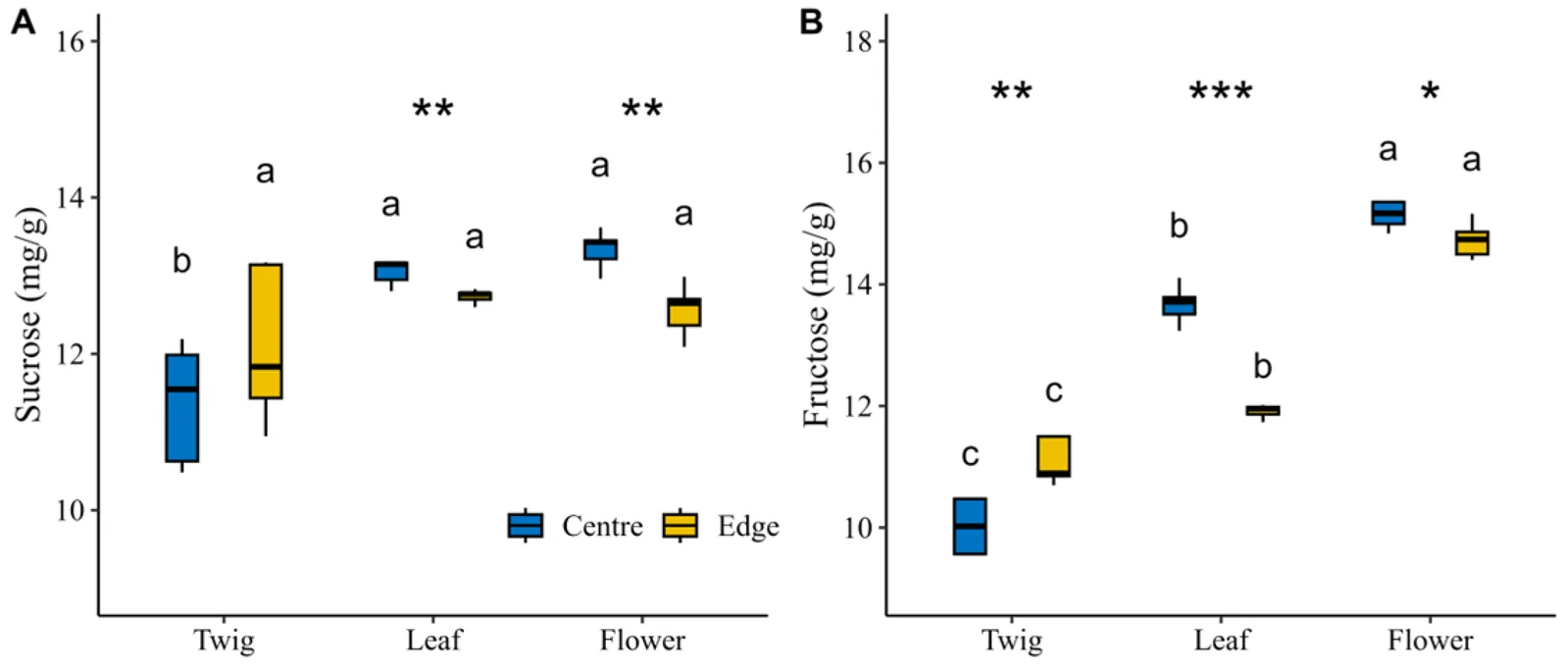
Sucrose and fructose concentrations in twigs, leaves, and flowers of *Salsola collina* at patch centers and edges: (**A**) sucrose (SUC) and (**B**) fructose (FRU). Note: Data are based on five plot-level replicates (*n* = 5). Lowercase letters compare organs within the same patch position. Groups sharing at least one letter (e.g., a and b) do not differ significantly, whereas groups with no letters in common differ significantly (*p* < 0.05). Asterisks indicate significant differences between patch centers and edges within the same organ: one, two, and three asterisks indicate * *p* < 0.05, ** *p* < 0.01, and *** *p* < 0.001, respectively. The absence of an asterisk indicates no significant center–edge difference.

**Figure 3:**
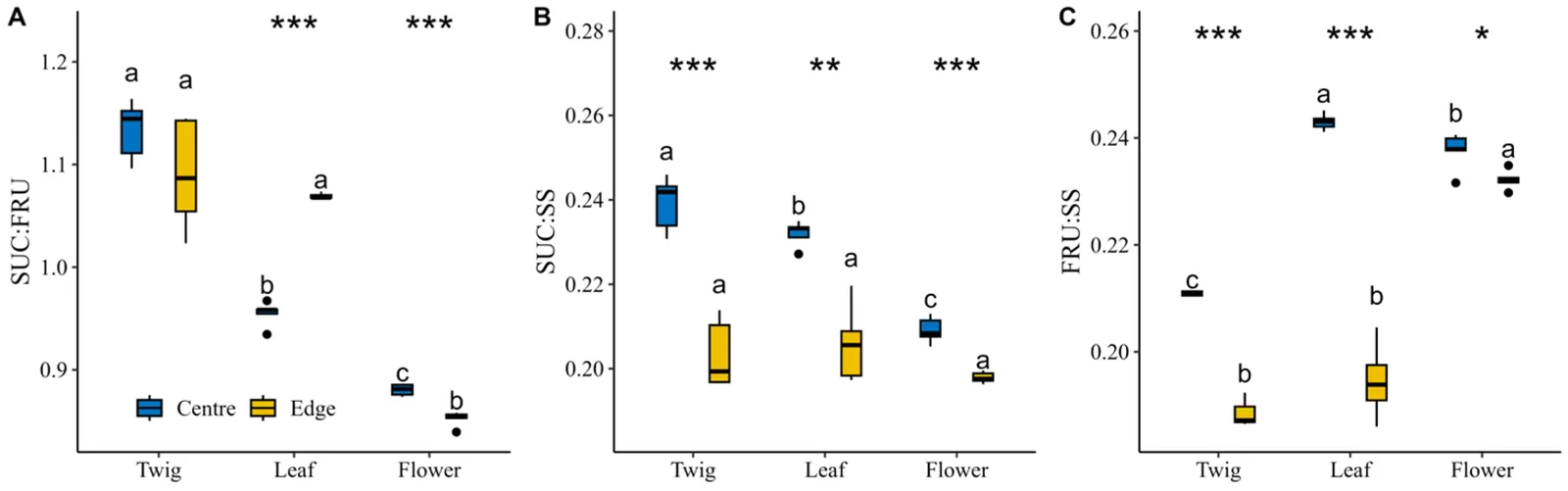
Carbohydrate component ratios in twigs, leaves, and flowers of *Salsola collina* at patch centers and edges: (**A**) sucrose-to-fructose ratio (SUC:FRU), (**B**) sucrose-to-total soluble sugar ratio (SUC:SS), and (**C**) fructose-to-total soluble sugar ratio (FRU:SS). Note: Data are based on five plot-level replicates (*n* = 5). Lowercase letters compare organs within the same patch position. Groups sharing at least one letter (e.g., a and b) do not differ significantly, whereas groups with no letters in common differ significantly (*p* < 0.05). Asterisks indicate significant differences between patch centers and edges within the same organ: one, two, and three asterisks indicate * *p* < 0.05, ** *p* < 0.01, and *** *p* < 0.001, respectively. The absence of an asterisk indicates no significant center–edge difference.

**Figure 4:**
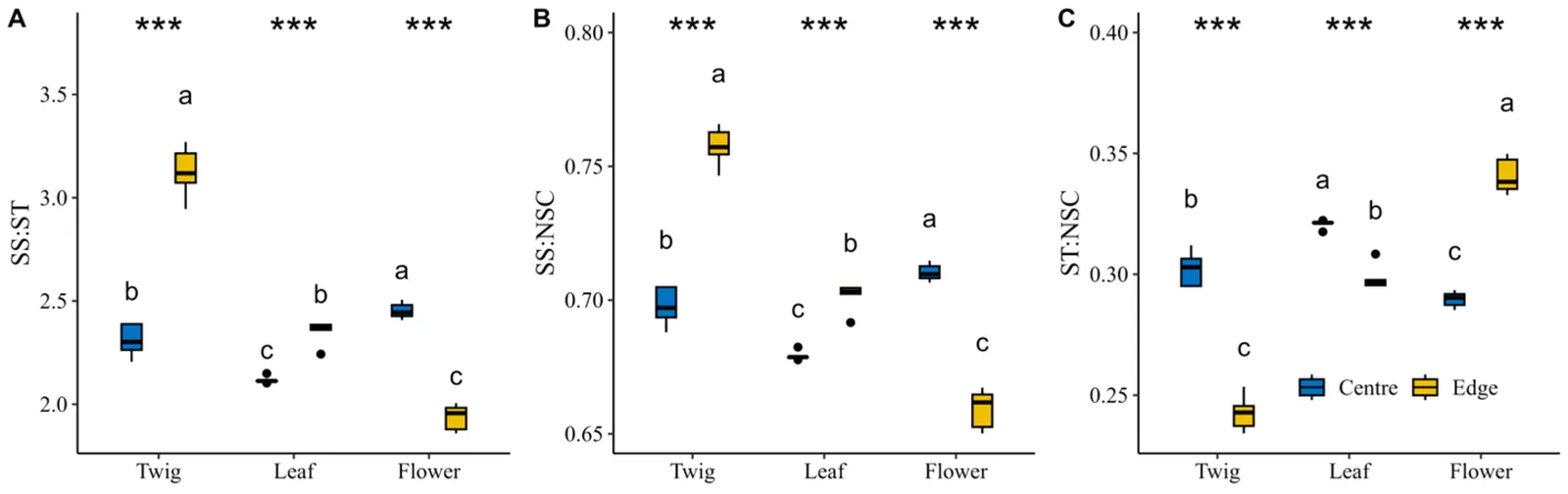
Carbohydrate component ratios in twigs, leaves, and flowers of *Salsola collina* at patch centers and edges: (**A**) total soluble sugar-to-starch ratio (SS:ST), (**B**) total soluble sugar-to-non-structural carbohydrate ratio (SS:NSC), and (**C**) starch-to-non-structural carbohydrate ratio (ST:NSC). Note: Data are based on five plot-level replicates (*n* = 5). Lowercase letters compare organs within the same patch position. Groups sharing at least one letter (e.g., a and b) do not differ significantly, whereas groups with no letters in common differ significantly (*p* < 0.05). Asterisks indicate significant differences between patch centers and edges within the same organ: three asterisks indicate *** *p* < 0.001. The absence of an asterisk indicates no significant center–edge difference.

**Figure 5:**
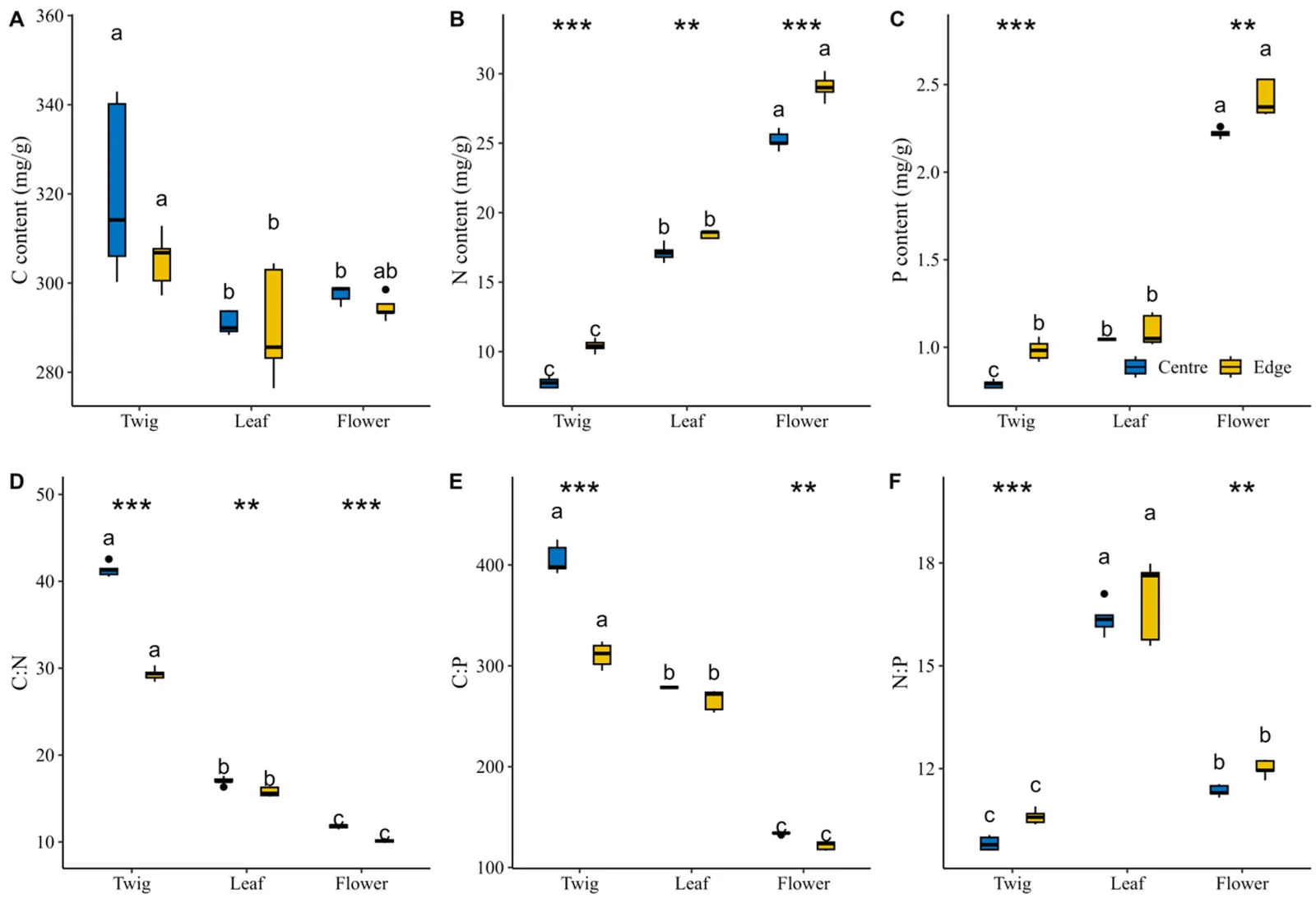
Carbon, nitrogen, and phosphorus concentrations and stoichiometric ratios in twigs, leaves, and flowers of *Salsola collina* at patch centers and edges: (**A**) carbon (C), (**B**) nitrogen (N), (**C**) phosphorus (P), (**D**) C:N, (**E**) C:P, and (**F**) N:P. Note: Data are based on five plot-level replicates (*n* = 5). Lowercase letters compare organs within the same patch position. Groups sharing at least one letter (e.g., a and ab) do not differ significantly, whereas groups with no letters in common differ significantly (*p* < 0.05). Asterisks indicate significant differences between patch centers and edges within the same organ: two and three asterisks indicate ** *p* < 0.01 and *** *p* < 0.001, respectively. The absence of an asterisk indicates no significant center–edge difference.

Two-way ANOVA revealed a significant main effect of organ type on all 17 traits examined (all *p* < 0.001; Table 1). Patch position significantly affected 14 of the 17 traits, whereas no significant main effect of patch position was detected for sucrose (SUC; *p* = 0.565), carbon (C; *p* = 0.091), or SUC:FRU (*p* = 0.139). Significant Patch × Organ interactions were detected for 14 traits, indicating that the magnitude and/or direction of center–edge differences varied among organs for these traits. In contrast, the interaction was not significant for non-structural carbohydrates (NSC; *p* = 0.186), C (*p* = 0.213), or N:P (*p* = 0.897). These interaction patterns were further examined using organ-specific center–edge comparisons (Figure 1, Figure 2, Figure 3, Figure 4 and Figure 5).

Patch × Organ interactions were significant for total soluble sugar (SS) and starch (ST) (both *p* < 0.001), but not for NSC (*p* = 0.186; Table 1; Figure 1). At patch centers, SS followed the order flowers > leaves > twigs, whereas at patch edges flowers had significantly higher SS than twigs, with leaves showing intermediate values. Compared with patch centers, SS was significantly higher at patch edges in twigs (*p* < 0.001) and leaves (*p* < 0.05), but not in flowers. ST was higher in leaves and flowers than in twigs at patch centers, whereas at patch edges it followed the order flowers > leaves > twigs; only flowers had significantly higher ST at the patch edge than at the center (*p* < 0.001). NSC consistently followed the order flowers > leaves > twigs at both patch positions and was significantly higher at patch edges in all three organs (*p* < 0.001 for twigs; *p* < 0.05 for leaves and flowers).

Sucrose and fructose both showed significant Patch × Organ interactions (*p* = 0.016 and *p* < 0.001, respectively; Table 1; Figure 2). At patch centers, sucrose concentrations were higher in leaves and flowers than in twigs, whereas no significant differences among organs were detected at patch edges. Compared with patch centers, sucrose concentrations were significantly lower at patch edges in leaves and flowers (both *p* < 0.01), whereas no significant center–edge difference was detected in twigs. Fructose concentrations followed the same organ ranking at both patch positions (flowers > leaves > twigs), but were significantly higher at patch edges in twigs (*p* < 0.01) and significantly lower in leaves (*p* < 0.001) and flowers (*p* < 0.05).

Significant Patch × Organ interactions were detected for SUC:FRU, SUC:SS, and FRU:SS (all *p* < 0.001; Table 1; Figure 3). At patch centers, SUC:FRU and SUC:SS followed the order twigs > leaves > flowers, whereas FRU:SS followed the order leaves > flowers > twigs. At patch edges, SUC:FRU did not differ significantly between twigs and leaves, and both were higher than in flowers; SUC:SS did not differ significantly among organs; and FRU:SS was higher in flowers than in twigs and leaves. Compared with patch centers, SUC:FRU was significantly higher at patch edges in leaves but lower in flowers (both *p* < 0.001), with no significant center–edge difference in twigs. SUC:SS was significantly lower at patch edges in all three organs (*p* < 0.001 for twigs and flowers; *p* < 0.01 for leaves), as was FRU:SS (*p* < 0.001 for twigs and leaves; *p* < 0.05 for flowers).

All three ratios showed significant Patch × Organ interactions (all *p* < 0.001; Table 1; Figure 4). At patch centers, SS:ST and SS:NSC followed the order flowers > twigs > leaves, whereas ST:NSC followed the order leaves > twigs > flowers. At patch edges, the corresponding rankings were twigs > leaves > flowers for SS:ST and SS:NSC, and flowers > leaves > twigs for ST:NSC. Compared with patch centers, SS:ST and SS:NSC were significantly higher at patch edges in twigs and leaves but lower in flowers, whereas ST:NSC was significantly lower at patch edges in twigs and leaves but higher in flowers (all *p* < 0.001).

Elemental concentrations and stoichiometric ratios varied among organs and patch positions (Table 1; Figure 5). Significant Patch × Organ interactions were detected for N, P, C:N, and C:P (all *p* ≤ 0.018), but not for C or N:P. N concentrations followed the order flowers > leaves > twigs at both patch positions. P concentrations followed the same order at patch centers, whereas at patch edges flowers had higher P concentrations than twigs and leaves, which did not differ significantly. Across patch positions, N concentrations were approximately 7–10, 17–18, and 25–29 mg g^−1^, and P concentrations were approximately 0.8–1.0, 1.0–1.1, and 2.2–2.4 mg g^−1^ in twigs, leaves, and flowers, respectively. N concentrations were significantly higher at patch edges in all organs, whereas P concentrations were significantly higher in twigs and flowers only. C:N and C:P followed the order twigs > leaves > flowers at both patch positions; C:N was significantly lower at patch edges in all organs, whereas C:P was significantly lower in twigs and flowers only. N:P was higher at patch edges (*p* = 0.003), with no significant Patch × Organ interaction (*p* = 0.897).

### 2.2. Organ-Specific Patterns of Center–Edge Trait Differences

**Figure 6:**
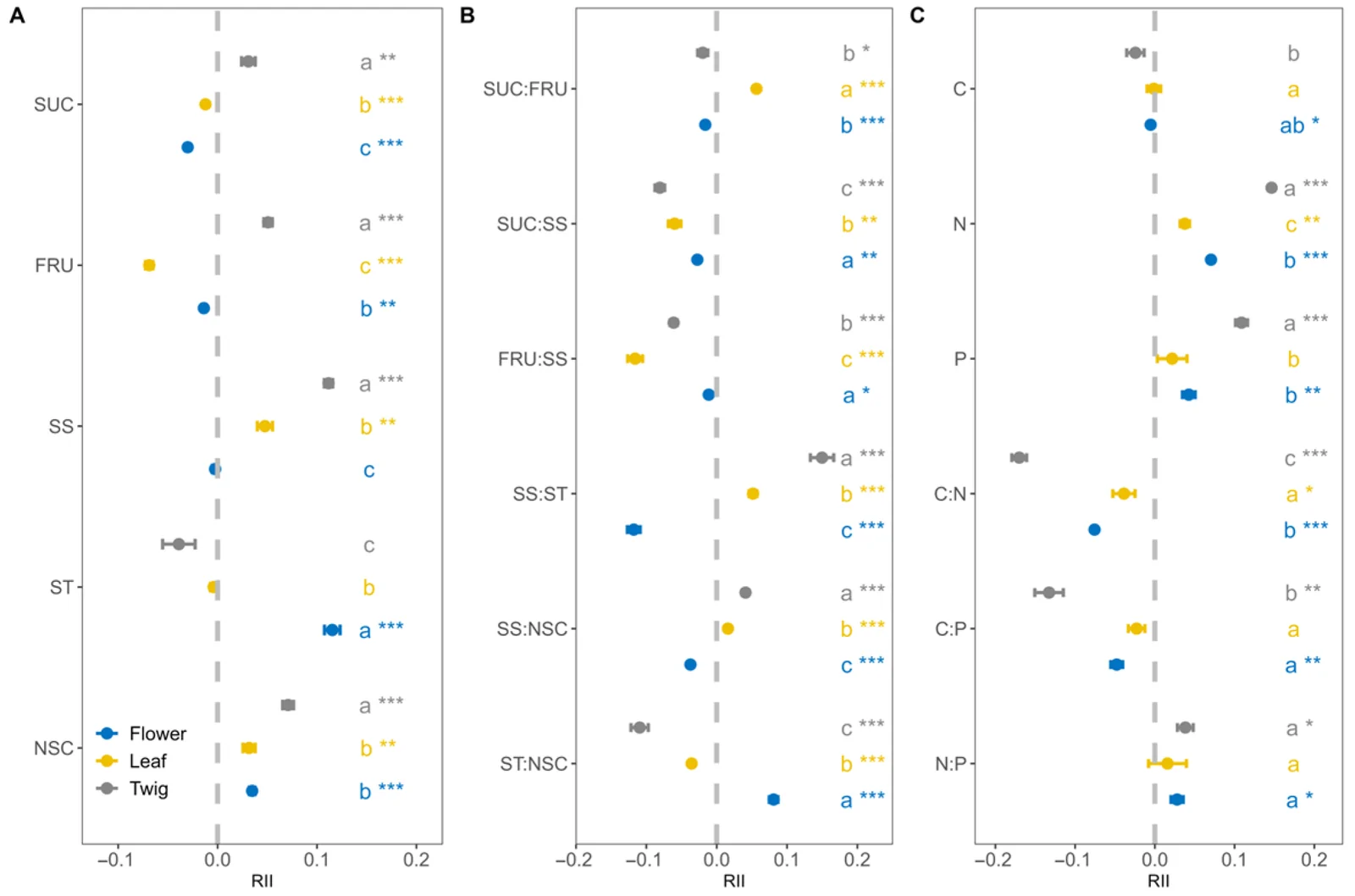
Relative interaction index (RII) values of plant traits in twigs, leaves, and flowers of *Salsola collina* between patch centers and edges: (**A**) non-structural carbohydrate traits, (**B**) carbohydrate ratios, and (**C**) elemental and stoichiometric traits. Note: Data are based on five plot-level replicates (*n* = 5). Flower, Leaf, and Twig represent flowers, leaves, and twigs, respectively. Different lowercase letters indicate significant differences among organs within the same patch position (*p* < 0.05). SUC, FRU, SS, ST, and NSC denote sucrose, fructose, total soluble sugars, starch, and non-structural carbohydrates, respectively; C, N, and P denote carbon, nitrogen, and phosphorus, respectively. The dashed line indicates RII = 0. Positive values indicate higher trait values at the patch edge than at the center, whereas negative values indicate lower values. Larger absolute RII values indicate greater relative center–edge differences. Asterisks indicate significant center–edge differences in the original trait values within the same organ: one, two, and three asterisks indicate * *p* < 0.05, ** *p* < 0.01, and *** *p* < 0.001, respectively. RII values were used descriptively and were not independently tested for significance.

Relative interaction index (RII) analysis summarized the direction and relative magnitude of center–edge differences in the measured traits of twigs, leaves, and flowers of *S. collina* (Figure 6). Non-structural carbohydrate (NSC) concentrations showed positive RII values in all three organs, indicating higher values at patch edges. Total soluble sugar (SS) also showed positive RII values in twigs and leaves, but values close to zero in flowers, whereas starch (ST) showed negative RII values in twigs, values close to zero in leaves, and positive values in flowers. Sucrose (SUC) and fructose (FRU) generally showed contrasting center–edge patterns among organs, with positive or near-zero RII values in twigs and negative values in leaves and flowers. Among carbohydrate ratios, SS:ST and SS:NSC showed positive RII values in twigs and leaves but negative values in flowers. For elemental and stoichiometric traits, N, P, and N:P generally showed positive RII values, whereas C:N and C:P showed negative values. Carbon (C) showed relatively small center–edge differences overall, with RII values close to zero across organs.

**Figure 7:**
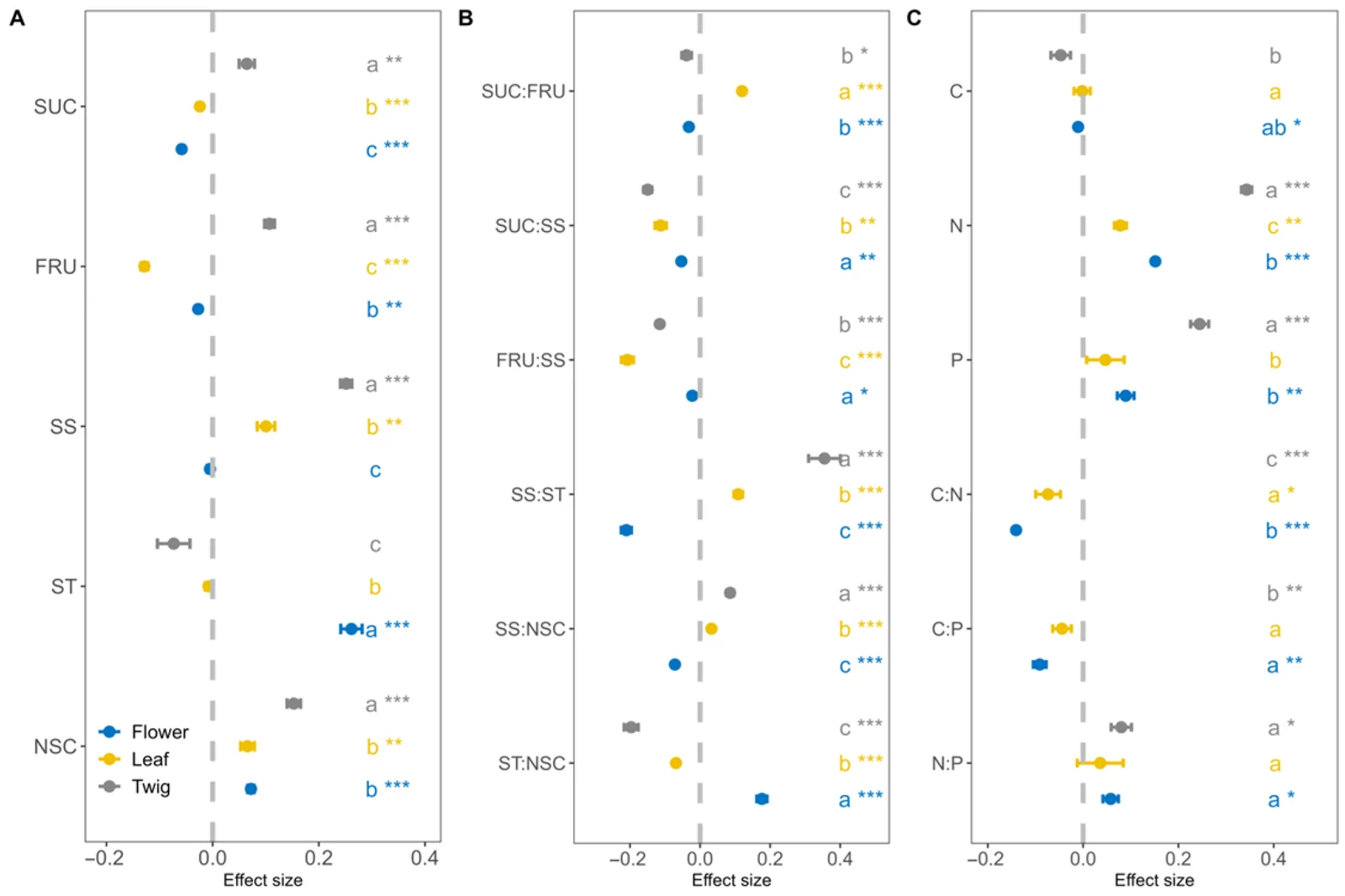
Relative edge-effect sizes of plant traits in twigs, leaves, and flowers of *Salsola collina* between patch centers and edges: (**A**) non-structural carbohydrate traits, (**B**) carbohydrate ratios, and (**C**) elemental and stoichiometric traits. Note: Data are based on five plot-level replicates (*n* = 5). Flower, Leaf, and Twig represent flowers, leaves, and twigs, respectively. Different lowercase letters indicate significant differences among organs within the same patch position (*p* < 0.05). SUC, FRU, SS, ST, and NSC denote sucrose, fructose, total soluble sugars, starch, and non-structural carbohydrates, respectively; C, N, and P denote carbon, nitrogen, and phosphorus, respectively. The dashed line indicates a relative edge-effect size of 0. Positive values indicate higher trait values at the patch edge than at the center, whereas negative values indicate lower values. Larger absolute effect-size values indicate greater proportional center–edge differences. Asterisks indicate significant center–edge differences in the original trait values within the same organ: one, two, and three asterisks indicate * *p* < 0.05, ** *p* < 0.01, and *** *p* < 0.001, respectively. Relative edge-effect sizes were used descriptively and were not independently tested for significance.

The relative edge-effect sizes summarized the magnitude and direction of center–edge differences in traits within each organ (Figure 7). Twigs generally showed larger absolute effect sizes than leaves and flowers for total soluble sugars, NSC, N, C:N, and C:P, with positive values for the first three traits and negative values for the latter two stoichiometric ratios. In leaves, sucrose and fructose showed negative effect sizes, accompanied by a positive value for SUC:FRU and negative values for SUC:SS and FRU:SS. In flowers, starch showed a positive effect size, whereas SS:ST and SS:NSC showed negative values.

### 2.3. Overall Variation Patterns of Plant Traits Across Organs and Patch Positions

**Figure 8:**
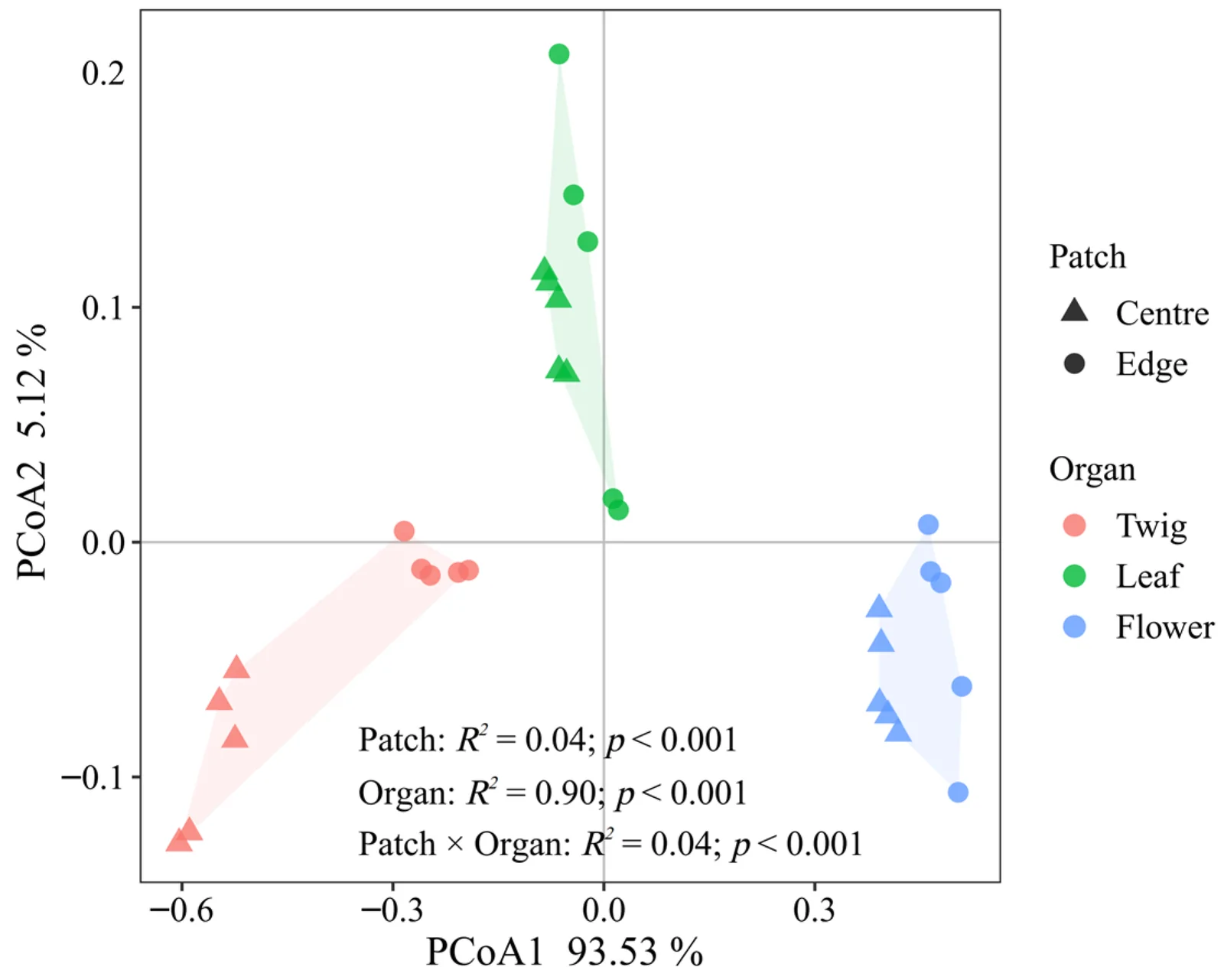
Principal coordinate analysis (PCoA) of multivariate trait variation in twigs, leaves, and flowers of *Salsola collina* at patch centers and edges. Note: The PCoA was based on seven non-redundant measured traits—C, N, P, sucrose (SUC), fructose (FRU), total soluble sugars (SS), and starch (ST)—after z-score standardization. Triangles and circles represent patch-center and patch-edge samples, respectively. Colors indicate organ type, and shaded polygons enclose samples of the same organ. The percentages on the axes indicate the proportions of variation explained by the first two coordinate axes. PERMANOVA results for Patch, Organ, and Patch × Organ are shown in the figure.

Principal coordinate analysis (PCoA) based on seven non-redundant measured traits showed a clear separation of twigs, leaves, and flowers along the first two axes (Figure 8). PCoA1 and PCoA2 explained 93.53% and 5.12% of the total variation, respectively. PERMANOVA indicated significant effects of patch position (*R*^2^ = 0.04), organ type (*R*^2^ = 0.90), and their interaction (*R*^2^ = 0.04) on multivariate trait variation (all *p* < 0.001), with organ type accounting for the largest proportion of variation.

**Figure 9:**
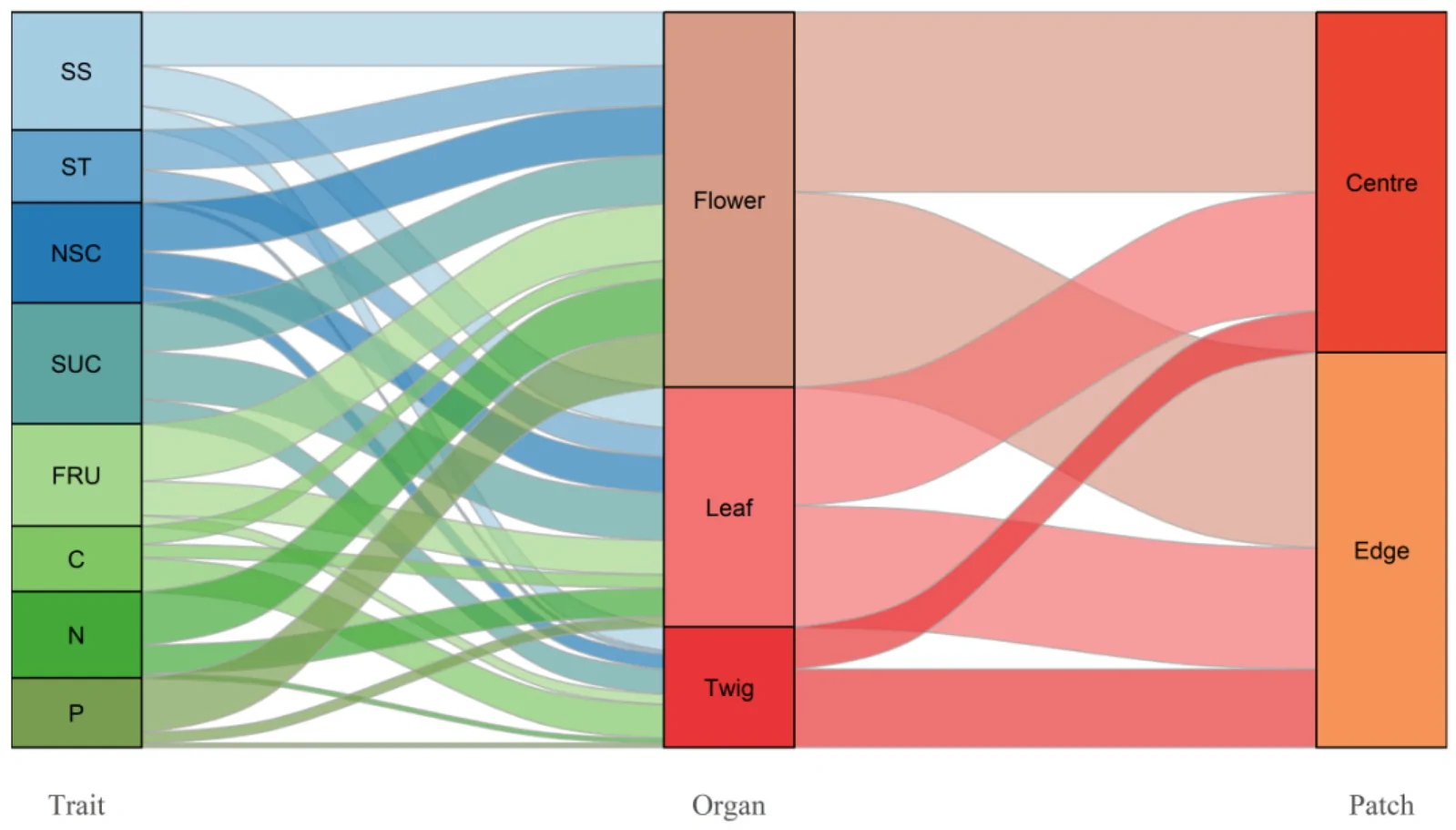
Sankey diagram of measured plant traits across organs and patch positions in *Salsola collina.* Note: SS, ST, NSC, SUC, and FRU represent total soluble sugars, starch, non-structural carbohydrates, sucrose, and fructose, respectively; C, N, and P represent carbon, nitrogen, and phosphorus, respectively. Twig, Leaf, and Flower denote the three organ types, whereas Centre and Edge denote patch centers and edges, respectively. Connections link the measured traits to organ types and patch positions. Flow widths represent the relative contributions of the measured trait values and should not be interpreted as statistical effect sizes or significance. Similar flow widths between an organ and the two patch positions do not necessarily indicate small center–edge differences in individual traits.

The Sankey diagram provided a descriptive overview of the measured carbohydrate and elemental traits across organs and patch positions in *S. collina* (Figure 9). The connections among traits, organs, and patch positions visually summarized the patterns presented in the preceding analyses.

## 3. Discussion

### 3.1. Organ-Specific Variation in Ecological Stoichiometry and Non-Structural Carbohydrates of S. collina

Organ type had a significant main effect on all traits examined in *S. collina* (Table 1) and accounted for the largest proportion of multivariate trait variation (Figure 8), indicating marked differences in tissue composition among twigs, leaves, and flowers. These differences are consistent with the contrasting structural, photosynthetic, and reproductive roles of the organs. Previous studies have documented organ-related variation in N and P traits [9,10], including differences among organs in *Calligonum* and *Artemisia* species [11,12]. Nutrient relationships among organs have also been examined in tropical dry forests [20]. This literature provides a context for the observed organ contrasts, which should be distinguished from differences associated with patch position.

At both patch centers and edges, N concentrations followed the order flowers > leaves > twigs, whereas C:N and C:P ratios showed the reverse pattern (Figure 5). P concentrations followed this organ ranking at patch centers; at patch edges, flowers had higher P concentrations than leaves and twigs, which did not differ significantly. Higher N and P concentrations in flowers may be associated with reproductive development, consistent with variation in nutrient concentrations among reproductive stages and organs reported for *Calligonum* species [11]. However, tissue concentrations do not directly quantify nutrient demand or the amount of nutrients allocated to reproduction. The higher C:N and C:P ratios in twigs indicate lower N and P concentrations relative to C, a pattern consistent with their structural role. Studies of woody tissues have likewise documented variation in nutrient concentrations and stoichiometric relationships [13,21]. All samples were collected during the full-flowering period; however, potential differences in flower age or developmental stage could still contribute to the observed organ contrasts. Without comparisons across reproductive stages, the higher nutrient concentrations observed in flowers cannot be assumed to persist throughout development or represent a fixed resource-use strategy.

NSC concentrations were higher in flowers than in leaves and twigs at both patch positions (Figure 1), whereas soluble sugars and starch did not consistently follow the same organ ranking. These patterns indicate differences in carbohydrate composition as well as total NSC concentration. An organ-level meta-analysis has shown that NSC and its components respond differently to nutrient enrichment [15]. Studies across grassland environments have also documented variation in NSC composition [22], while leaf and root NSC responses to nitrogen addition can differ [23]. Such findings provide a broader context for the carbohydrate variability observed here but do not establish the processes responsible for these patterns. Because organ biomass was not measured, higher NSC concentrations cannot be interpreted as larger whole-organ NSC pools. Determining whether these concentration differences correspond to differences in carbon allocation, turnover, or storage requires additional measurements of biomass and carbon fluxes.

### 3.2. Patch-Position Differences in Ecological Stoichiometry and Non-Structural Carbohydrates Among Organs of S. collina

Patch position was associated with differences in several nutrient and carbohydrate traits of *S. collina*. N concentrations were higher at patch edges in all three organs, whereas P concentrations were higher in twigs and flowers, with no significant center–edge differences in C concentrations (Figure 5). Accordingly, C:N ratios were lower at edges in all organs, C:P ratios were lower at edges in twigs and flowers, and N:P was higher at edges overall. The lower C:N and C:P ratios primarily reflected higher tissue N and P concentrations rather than lower C concentrations. Studies of vegetation patches have documented spatial variation in soil properties [4,24], while research on plant spatial patterns has considered relationships with local environmental conditions and species interactions [25]. These studies provide ecological context for the observed center–edge differences, including potential variation in resource availability and neighboring vegetation. However, because soil moisture, nutrient availability, and microclimate were not measured concurrently, the observed nutrient patterns cannot be attributed directly to particular environmental conditions at patch edges.

Although NSC concentrations were higher at edges in all three organs, soluble sugars and starch showed different center–edge patterns among organs (Figure 1). Twigs and leaves had higher soluble sugar concentrations at edges without significant differences in starch, whereas flowers had higher starch concentrations without a significant difference in soluble sugars. SS:ST and SS:NSC ratios were correspondingly higher at edges in twigs and leaves but lower in flowers (Figure 4). Because these ratios are mathematically derived from the same measured carbohydrate variables, they are interpreted here as complementary descriptors of carbohydrate composition rather than as independent physiological evidence. These patterns indicate a greater soluble sugar fraction in edge twigs and leaves and a greater starch fraction in edge flowers, rather than demonstrating temporal starch accumulation. Drought experiments have reported differing responses of soluble sugars and starch under varying stress conditions [17,18,19]. Water and nitrogen treatments can also influence carbon assimilation and NSC concentrations [26], while environmental stress and developmental stage may affect relationships between carbohydrates and stoichiometric traits [27]. These studies provide physiological context, but the present center–edge comparison does not establish a drought or nutrient gradient or demonstrate distinct carbohydrate regulation strategies.

RII and relative edge-effect sizes provided descriptive summaries of these spatial contrasts (Figure 6 and Figure 7). Twigs had numerically larger absolute RII values and relative edge-effect sizes than leaves and flowers for total soluble sugars, NSC, N, C:N, and C:P. These indices describe the direction and magnitude of differences, rather than identifying their causal drivers. Organ-dependent trait variation has also been reported along aridity gradients [28], while drought studies of *S. collina* [6] and other species [29] illustrate physiological responses under defined stress conditions. These studies provide context for possible processes that warrant further investigation but do not establish that the same processes explain variation within natural patches.

The nutrient patterns observed here can be considered in the broader context of variation in plant C–N–P stoichiometry across Chinese drylands [30], including associations with soil conditions in Xinjiang halophytic shrubs [31] and with abiotic and biotic factors along desert–grassland gradients [32]. Stoichiometric variation has also been reported along salinity gradients [33,34] and in relation to altitude, grazing, temporal variation, and nutrient inputs [35,36,37]. At the organ level, previous studies have examined stoichiometric homeostasis, altitudinal variation, and nutrient distribution among desert plant organs [38,39,40,41], while nitrogen-addition and drought–rewatering experiments have shown that NSC and C:N:P traits can vary under altered resource conditions [42,43]. These studies provide broader ecological context for the present findings but do not identify the drivers of the observed center–edge differences.

For carbohydrate traits, variation in NSC among organs and biomes [44] and its relationships with plant economic traits [45,46] indicate that differences in NSC concentration should not be assigned a single functional interpretation. Drought experiments [47,48], contrasting water and nitrogen supplies [49], and studies examining groundwater depth and plant age in *Haloxylon ammodendron* [50] further illustrate that both environmental and developmental factors can influence carbohydrate traits. Distinguishing the relevance of these factors to *S. collina* requires direct measurements of local environmental conditions and developmental stage.

Concurrent measurements of soil moisture, nutrient availability, light, and microclimate would help assess their relationships with the observed tissue nutrient and carbohydrate patterns. Combining these measurements with organ biomass, growth, and reproductive development would allow concentration differences to be interpreted in the context of whole-organ nutrient and carbon pools. Repeated sampling across developmental stages would further clarify whether the center–edge patterns persist over time or vary with phenology. In addition, because plants occurring outside clearly defined patches were not included in the present sampling design, future studies should incorporate non-patch plants as an additional reference group to determine whether the observed center–edge patterns are specific to established patches or extend to plants growing outside them.

## 4. Materials and Methods

### 4.1. Study Area

The study was conducted in Mulei Kazak Autonomous County, Xinjiang Uygur Autonomous Region, northwestern China (approximately 43°46′–45°38′ N, 89°08′–91°28′ E). The region has a typical temperate continental arid climate, with a mean annual temperature of approximately 5–6 °C and a mean annual precipitation of approximately 344 mm [51]. A previous field study conducted in Mulei County recorded a total rainfall of 135.80 mm from 24 April to 15 September 2020 [51]. The study focused on *S. collina*, which forms distinct vegetation patches in the area. Field surveys and sample collection were conducted in areas characterized by continuous *S. collina* distribution, minimal human disturbance, and well-developed vegetation patches.

### 4.2. Sample Collection and Processing

Five 10 m × 10 m plots were established within the study area, with at least 20 m between plots. Within each plot, three well-developed *S. collina* patches of similar canopy size (approximately 3.5–4.0 m in diameter) and growth condition were selected, with at least 3 m between neighboring patches. Sampling was restricted to well-developed *S. collina* patches; plants occurring outside clearly defined patches were not included in the sampling design. The patch center was defined as the point farthest from the live canopy margin, whereas the edge was defined as a 50-cm-wide band immediately inside the canopy margin at the boundary between the vegetated patch and surrounding bare soil. Depending on patch size, the distance between center and edge sampling locations was approximately 1.5–2.0 m.

Sampling was conducted in the morning in July 2021 during the full-flowering period of *S. collina*. At both the center and edge of each patch, 2–3 similarly developed twigs were collected from each of four directions (north, east, south, and west), with the attached leaves and flowers kept intact. Materials from the four directions within the same patch and position were pooled, after which twigs, leaves, and flowers were separated to form one composite sample for each organ. Individual plant identity was not recorded during field sampling; therefore, the resulting composite samples were defined at the patch × position level rather than at the individual-plant level.

Samples were placed in labeled paper envelopes and transported to the laboratory, where heat treatment was initiated within approximately 1 h of collection. Plant materials were treated at 105 °C for 30 min [52] and then dried at 70 °C to constant weight. The dried samples were ground into homogeneous powders and stored in sealed bags until chemical analysis. The three patches within each plot were treated as subsamples, and their measurements at the same patch position were averaged at the plot level. Thus, each organ × patch-position combination comprised five independent plot-level replicates (*n* = 5), with center and edge observations paired within plots.

### 4.3. Sample Measurements

#### 4.3.1. Determination of Plant C, N, and P Concentrations

Plant carbon (C) concentration was determined using the potassium dichromate–sulfuric acid oxidation method [52]. Briefly, 0.20 g of plant powder was mixed with 5 mL of 0.8 mol L^−1^ potassium dichromate solution (1/6 K_2_Cr_2_O_7_) and 5 mL of concentrated sulfuric acid, and digested in an oil bath at 170–180 °C for 5 min. After cooling, the digest was transferred to a 250 mL conical flask with distilled water and adjusted to approximately 50–60 mL. Three drops of o-phenanthroline indicator were added, and the solution was titrated with standard ferrous sulfate solution to a reddish-brown endpoint. C concentration was calculated from the titration results.

Plant nitrogen (N) and phosphorus (P) concentrations were determined after sulfuric acid–hydrogen peroxide (H_2_SO_4_–H_2_O_2_) wet digestion [52,53]. Approximately 0.20 g of plant powder was digested with 5 mL of concentrated H_2_SO_4_–H_2_O_2_ mixture until the digest became clear and colorless, and was subsequently diluted to 100 mL with distilled water. For N determination, an aliquot of the digest was analyzed using the Kjeldahl method. After addition of excess 40% NaOH, the released ammonia was distilled and absorbed in 2% boric acid containing methyl red–bromocresol green indicator. The solution was then titrated with standard hydrochloric acid to a stable light-purple endpoint. Blank samples were analyzed in parallel, and N concentration was calculated from the difference in acid consumption between the sample and blank.

For P determination, a 5 mL aliquot of the same H_2_SO_4_–H_2_O_2_ digest was transferred to a 50 mL volumetric flask. After addition of 2 mL of 2,6-dinitrophenol indicator, the solution was neutralized with 6 mol L^−1^ NaOH until a faint yellow color developed. Subsequently, 10.00 mL of ammonium vanadomolybdate reagent was added, and the solution was diluted to volume with distilled water. After standing for 15 min, absorbance was measured at 450 nm using a microplate reader (Multiskan FC, Thermo Fisher Scientific, Waltham, MA, USA), and P concentration was determined from a phosphorus standard curve [53].

#### 4.3.2. Determination of Non-Structural Carbohydrates

Approximately 0.050 g of dried plant powder was extracted with 1.5 mL of 80% ethanol in a boiling-water bath for 20 min. After cooling, the extract was centrifuged at 12,000 rpm for 10 min, and the supernatant was collected for the determination of total soluble sugars (SS), sucrose (SUC), and fructose (FRU). Hot 80% ethanol extraction followed established procedures for plant non-structural carbohydrate analysis [54].

Total soluble sugar concentration was determined using the anthrone colorimetric method [55]. Briefly, 0.01 mL of the extract was mixed with 0.19 mL of distilled water, 0.06 mL of anthrone reagent, and 0.50 mL of concentrated sulfuric acid, followed by incubation at 95 °C for 10 min. Absorbance was measured at 620 nm using a microplate reader (Multiskan FC, Thermo Fisher Scientific, Waltham, MA, USA), and SS concentration was calculated from a glucose standard curve. For sucrose determination, 0.01 mL of the extract was mixed with 0.05 mL of distilled water and 0.04 mL of 7.6 mol L^−1^ KOH and incubated at 95 °C for 10 min. After cooling, 0.72 mL of anthrone–sulfuric acid working solution was added, followed by a second incubation at 95 °C for 10 min. Absorbance was measured at 620 nm using the same microplate reader described above, and SUC concentration was calculated from a sucrose standard curve [56]. For fructose determination, 0.09 mL of the extract was mixed with 0.63 mL of 80% ethanol and 0.18 mL of resorcinol ethanol solution and incubated at 95 °C for 30 min. Absorbance was measured at 480 nm using the same microplate reader described above, and FRU concentration was calculated from a fructose standard curve [57].

Starch (ST) was determined using the residue remaining after soluble-sugar extraction. The residue was washed with 1.0 mL of 80% ethanol at 50 °C for 30 min and centrifuged at 10,000 rpm for 5 min, after which the supernatant was discarded. The residue was then mixed with 0.50 mL of distilled water and heated at 95 °C for 15 min to promote gelatinization. After cooling, 0.35 mL of hydrochloric acid was added, and the sample was extracted for 15 min. Subsequently, 0.85 mL of distilled water was added, and the mixture was centrifuged at 10,000 rpm for 10 min. For colorimetric determination, 0.03 mL of the resulting starch extract was mixed with 0.12 mL of distilled water and 0.75 mL of anthrone–sulfuric acid working solution and incubated at 95 °C for 10 min. Absorbance was measured at 620 nm using the same microplate reader described above, and ST concentration was calculated from a glucose standard curve [55].

Non-structural carbohydrate (NSC) concentration was calculated as:

NSC = SS + ST(1)

where SS and ST represent total soluble sugar and starch concentrations, respectively. The ratios SUC:FRU, SUC:SS, FRU:SS, SS:ST, SS:NSC, and ST:NSC were also calculated to describe the relative composition of carbohydrate components. Because these ratios were mathematically derived from the measured carbohydrate variables, they were interpreted as descriptive measures of carbohydrate composition rather than as independent physiological evidence.

### 4.4. Statistical Analysis

The plot was used as the independent experimental unit (*n* = 5), with the three patches within each plot averaged as subsamples. Because center and edge samples were obtained within the same plots, center–edge comparisons were treated as paired observations. For each trait, the effects of patch position (Patch), organ type (Organ), and their interaction (Patch × Organ) were evaluated using two-way ANOVA with plot included as a blocking factor. Normality of paired differences and homogeneity of variance were assessed using the Shapiro–Francia and Levene’s tests, respectively. When the Patch × Organ interaction was significant, center–edge differences within each organ were examined using paired t-tests with Holm-adjusted *p*-values. Differences among organs within each patch position were assessed using the least significant difference (LSD) test. Statistical significance was set at *p* < 0.05.

The relative interaction index (RII) and relative edge-effect size were calculated as descriptive measures of center–edge differences:

RII = (Xe − Xc)/(Xe + Xc)(2)

Effect size = (Xe − Xc)/Xc(3)

where Xe and Xc represent trait values at the patch edge and center, respectively. Positive values indicate higher trait values at the edge, whereas negative values indicate lower values. RII is bounded between −1 and 1 and symmetric around zero, facilitating comparisons among traits, whereas the relative edge–effect size expresses the proportional difference relative to the center value. Larger absolute values of either index indicate greater relative center–edge differences. Both indices were used descriptively rather than as independent inferential tests.

Multivariate analyses were based on seven non-redundant primary traits (C, N, P, SUC, FRU, SS, and ST); NSC and derived ratios were excluded to avoid mathematical redundancy. Traits were z-score standardized, and Euclidean distances were used for principal coordinate analysis (PCoA) and PERMANOVA. PERMANOVA included Patch, Organ, and Patch × Organ, with 999 permutations restricted within plots. Homogeneity of multivariate dispersion was assessed using PERMDISP. Analyses were performed using R software (version 4.5.1; R Core Team, Vienna, Austria) with the vegan package (version 2.7-1). Data are presented as mean ± standard error (SE).

## 5. Conclusions

The study showed that twigs, leaves, and flowers of *Salsola collina* differed in C:N:P stoichiometry and NSC profiles, with organ type accounting for the largest proportion of multivariate trait variation. Patch position was also associated with differences in nutrient and carbohydrate traits, and the significant Patch × Organ interaction indicated that center–edge differences in the multivariate trait profile varied among organs. N and NSC concentrations were higher at patch edges in all three organs, while soluble sugar concentrations were higher in edge twigs and leaves and starch concentrations were higher in edge flowers. These findings highlight organ-specific variation in nutrient and carbohydrate traits of *S. collina* across different positions within natural patches. Further studies incorporating soil properties, microclimatic conditions, plant biomass, and developmental stage would help clarify the environmental and physiological processes associated with these center–edge differences.

## Figures and Tables

**Table 1:** Two-way ANOVA effects of patch position, organ type, and their interaction on ecological stoichiometric characteristics and non-structural carbohydrate traits of *Salsola collina*.

Trait	Patch		Organ		Patch × Organ	
	*F*	*p*	*F*	*p*	*F*	*p*
SUC	0.341	0.565	15.215	<0.001	4.911	0.016
FRU	9.876	0.004	466.661	<0.001	48.615	<0.001
SS	52.603	<0.001	54.534	<0.001	19.838	<0.001
ST	17.985	<0.001	206.352	<0.001	43.307	<0.001
NSC	45.243	<0.001	108.289	<0.001	1.808	0.186
C	3.104	0.091	13.330	<0.001	1.650	0.213
N	152.548	<0.001	2444.246	<0.001	11.692	<0.001
P	42.742	<0.001	1598.866	<0.001	4.745	0.018
SUC:FRU	2.343	0.139	231.759	<0.001	28.766	<0.001
SUC:SS	126.555	<0.001	26.565	<0.001	10.849	<0.001
FRU:SS	337.747	<0.001	206.056	<0.001	81.031	<0.001
SS:ST	43.626	<0.001	158.166	<0.001	208.166	<0.001
SS:NSC	22.244	<0.001	153.119	<0.001	224.941	<0.001
ST:NSC	22.244	<0.001	153.119	<0.001	224.941	<0.001
C:N	660.249	<0.001	5795.582	<0.001	329.515	<0.001
C:P	148.394	<0.001	1686.215	<0.001	71.215	<0.001
N:P	11.343	0.003	393.283	<0.001	0.109	0.897

Note: SUC, sucrose; FRU, fructose; SS, total soluble sugars; ST, starch; NSC, non-structural carbohydrates; C, carbon; N, nitrogen; and P, phosphorus. Trait labels containing a colon indicate the ratio of the first component to the second (e.g., SUC:FRU, sucrose-to-fructose ratio; C:N, carbon-to-nitrogen ratio). Patch × Organ denotes the interaction between patch position and organ type.

## Data Availability

The data supporting the findings of this study are available from the corresponding author upon reasonable request.
